# Testing Club Convergence in Female Smoking Prevalence

**DOI:** 10.3389/fgwh.2022.875813

**Published:** 2022-07-11

**Authors:** Fabrizio Ferretti, Michele Mariani, Elena Sarti

**Affiliations:** Department of Communication and Economics, School of Social Sciences, University of Modena and Reggio Emilia, Reggio Emilia, Italy

**Keywords:** affordability, convergence, clubs, female smoking prevalence, ordered logit model

## Abstract

In this paper, we applied the concept of convergence to examine the evolution of smoking prevalence among women in 191 countries worldwide from 1990 to 2019. First, the non-linear time-varying factor model proposed by Phillips and Sul was adopted to identify potential clusters (clubs), wherein groups of countries converge to similar female smoking rates. Second, an ordered logit regression model was used to assess the impact of cigarette affordability on the probability of falling within a given cluster. The hypothesis of global convergence was rejected. However, the clustering algorithm successfully identified five and nine clubs, within countries with increasing and decreasing smoking prevalence, respectively. A higher relative income-price ratio (i.e., lower cigarette affordability) increased the likelihood of belonging to a club of countries with a low prevalence of female tobacco smoking.

## Introduction

Tobacco control is a global public health priority (1). Currently, about one-sixth of the worldwide non-communicable disease-related deaths are attributable to tobacco smoking (2). This is why one of the United Nations Sustainable Development targets to ensure healthy lives and promote wellbeing (i.e., Target 3*a*, Sustainable Development Goal 3) is to “Strengthen the World Health Organization's (WHO) Framework Convention on Tobacco Control” (3).

During the last two decades, progress has been made in implementing tobacco demand-reduction measures, such as reducing cigarette affordability through taxation, passing smoke-free laws, and banning advertising and sponsorship of tobacco products (4, 5). These efforts have been associated with a substantial reduction in the prevalence of smoking in many world regions (6). For instance, since 1990, the global age-standardized prevalence rate of smoking tobacco use in the male and female populations (aged 15 years and older) has decreased by 27.5 and 37.7%, respectively (7). However, despite these significant achievements, progress on tobacco cessation varies greatly across countries, and we are still very far from ending the global “tobacco epidemic” (8).

Given the considerable health and economic impact of tobacco use, a large body of literature has investigated smoking prevalence across different populations and over time (9). However, to the best of our knowledge, there is a lack of research on identifying potential convergence clubs, where groups of countries tend to converge to similar levels of smoking prevalence. This brief research paper attempts to fill this gap by analyzing club convergence in the prevalence of smoking tobacco use among the female population in 191 countries worldwide, during the time period 1990–2019. We approach this issue by applying the methodology developed by Phillips and Sul (10, 11) to the latest and comprehensive estimates of the female smoking prevalence provided by the Global Burden of Disease (GBD) 2019 Tobacco Collaborators (7). This methodology allows the clustering of countries into groups according to their tendency to convergence toward a common long-term value of the variable of interest (the age-standardized rate of smoking prevalence among women aged 15 years and older, for the purpose of this paper).

Slight differences have been observed in male smoking prevalence between developed and developing countries (12). Conversely, smoking habits among women differ substantially between countries and regions with different levels of socio-economics development (12, 13). This phenomenon motivated our focus on the female population. As smoking rates tend to decline in western nations, women in developing countries, especially in populous emerging economies, represent a new and promising market for the tobacco industry (14). In such contexts, an expansion of tobacco consumption would significantly increase the global burden of smoking-related diseases. Thus, analyzing the potential processes of convergence in female smoking prevalence between countries with different levels of development may be useful in implementing effective tobacco control policies (15).

## Data and Methods

We analyzed the evolution of the prevalence of smoking tobacco use among women in a large number of countries worldwide in order to address whether or not smoking rates tend to converge over time. For this purpose, we applied the concept of convergence (16) to the female smoking prevalence, using data from a recent publicly available database developed within the GBD Study (7). This database contains estimates of the age-standardized smoking prevalence rates by gender (in people aged 15 years and older) across 191 countries between 1990 and 2019 (the dataset is publicly available at: http://ghdx.healthdata.org/record/ihme-data/gbd-2019-smoking-tobacco-use-prevalence-1990-2019).

The concept of convergence has been widely utilized to examine the long-term evolution of economic indicators—e.g., the gross domestic product (GDP) or health care expenditures—between countries or regions (17, 18). More recently, convergence analysis has been applied to the study of the spread of non-communicable disease risk factors, such as overweight and obesity rates (19, 20). This literature is usually based on the non-linear time-varying factor model proposed by Phillips and Sul (10). The model allows testing the null hypothesis of convergence between a given set of countries, and if all countries do not converge toward a common value, it looks to identify potential patterns of convergence at the subset (or “club”) level (11).

To briefly outline the methodology of Phillips and Sul (10), let us denote with *FSP*_*it*_ the female smoking prevalence in country *i* and year *t*. We recognize in *FSP*_*it*_ the presence of a common (*g*_*it*_) and an idiosyncratic (*a*_*it*_) component, so that *FSP*_*it*_ = *g*_*it*_ + *a*_*it*_. As we aimed to examine the evolution over time of the idiosyncratic component, *FSP*_*it*_ can be transformed such that the common and idiosyncratic components are separated as follows:


(1) $$\begin{array}{r} {FSP}_{it}=\left(\frac{g_{it}\;+\;a_{it}}{\mu_{it}}\right)\mu_{t}=\;\delta_{it}\mu_{t} \end{array}$$

In equation (1), μ_*t*_ is the common trend component across countries, and δ_*it*_ is a time-varying heterogeneous component [measuring the heterogenous distance between μ_*t*_ and *FSP*_*it*_ (20)]. In other words, δ_*it*_ denotes the deviation of the female smoking prevalence in country *i* from the common path (i.e., the trend component μ_*t*_). In order to remove the common factor, the relative transition parameter (*h*_it_) and its cross-sectional variation (*H*_*it*_) are defined as follows:


(2) $$\begin{array}{l} h_{it}=\frac{FSP_{it}}{N^{-1}\sum_{i=1}^{N}FSP_{it}}=\frac{\delta_{it}}{N^{-1}\sum_{i=1}^{N}\delta_{it}} \end{array}$$

(3) $$\begin{array}{r} H_{it}=N^{-1}\sum_{i=1}^{N}{\left(h_{it}-1\right)}^{2}\;\to \;0,\;as\;t\;\to \;\infty \end{array}$$

This methodology assumes that, at some future point in time, countries will converge to a long-term steady-state (that is, lim_t → ∞_ δ_*it*_ = δ_*i*_ = δ, for all *i* = 1, …, *N*), although their paths may differ significantly. Thus, if the component δ_*it*_ tends to converge toward δ, there is evidence in favor of the convergence hypothesis. In this case, *h*_*it*_ would tend to 1 and *H*_*it*_ to 0, as time tends to infinity (19). Phillips and Sul (10, 11) suggest the use of a simple time-series regression to test the hypothesis of convergence. This so-called *log*-*t* regression is defined as:


$$\begin{array}{r} \log\frac{H_{1}}{H_{t}}\;-2log\left[log\left(t\right)\right]=\alpha +\beta log\left(t\right) \end{array}$$

(4) $$\begin{array}{r} +\nu_{t},\;t=\left[rT\right]+\;1,\ldots ,T \end{array}$$

[where *r* is usually set equal to 0.3 (10)]. The test involves a robust one-sided *t*-test of the inequality of the null hypothesis using a heteroskedasticity and autocorrelation consistent standard error (HAC). At the 5% level, the null hypothesis of convergence H_0_: δ_i_ = δ (i.e., β = 0) is rejected if the conventional t-statistic takes values lower than −1.65 (10).

Identifying factors driving club formation is outside the scope of this brief research report. However, we attempted to evaluate the role of affordability (i.e., the ability of consumers to purchase cigarettes). Earlier studies (21, 22) have demonstrated a strong and consistent association between affordability and consumption. In this paper, affordability was defined by the cigarette relative income price (*RIP*). Specifically, *RIP* is the ratio between price and income (21) and is measured by the percentage of per capita GDP required to buy 100 packs of cigarettes. Thus, the higher this ratio, the less affordable cigarettes are, and vice versa.

To assess the impact of cigarettes affordability on the probability of country *i* to falls within a given cluster of countries—i.e., a given club—we estimated the following ordered logit model:


(5) $$\begin{array}{r} logit\;FSP_{ct}=\alpha_{c}+\beta^{\prime }RIP_{i}\;and\;c=1,2,\ldots ,\;C-1 \end{array}$$

In equation (5), the dependent variable is an ordinal response variable, with *C* categories ranked in descending order (according to the different clubs identified by Phillips and Sul's methodology), and cigarette affordability is measured by its initial value. The model is thus defined by a set of *C*-1 equations, where the parameters α_*c*_ are the thresholds (or cut-points) levels. Here, a positive and statistically significant coefficient for the variable *RIP* indicates an increased chance that a country with a higher relative income-price ratio (i.e., lower affordability) will be in a cluster with a lower smoking prevalence. Data on cigarette affordability were collected from the WHO Global Health Observatory (23) and integrated with the estimates provided by Blecher and Walbeek (24).

All analyses were performed in Stata version 16.1 (Stata Corp LLC, TX, USA). This methodology aims to test for convergence among countries (and not simply to cluster countries). Thus, we used the package *psecta*, developed by Du (25), to analyze the processes of convergence and the corresponding algorithm added by Phillips and Sul (11) to test for convergence, implement club convergence, and make the related analysis. Finally, given the use of smooth (i.e., modeled) time series, there was no need to separate data on smoking prevalence into a trend and a cyclical component.

## Results

**Figure 1:**
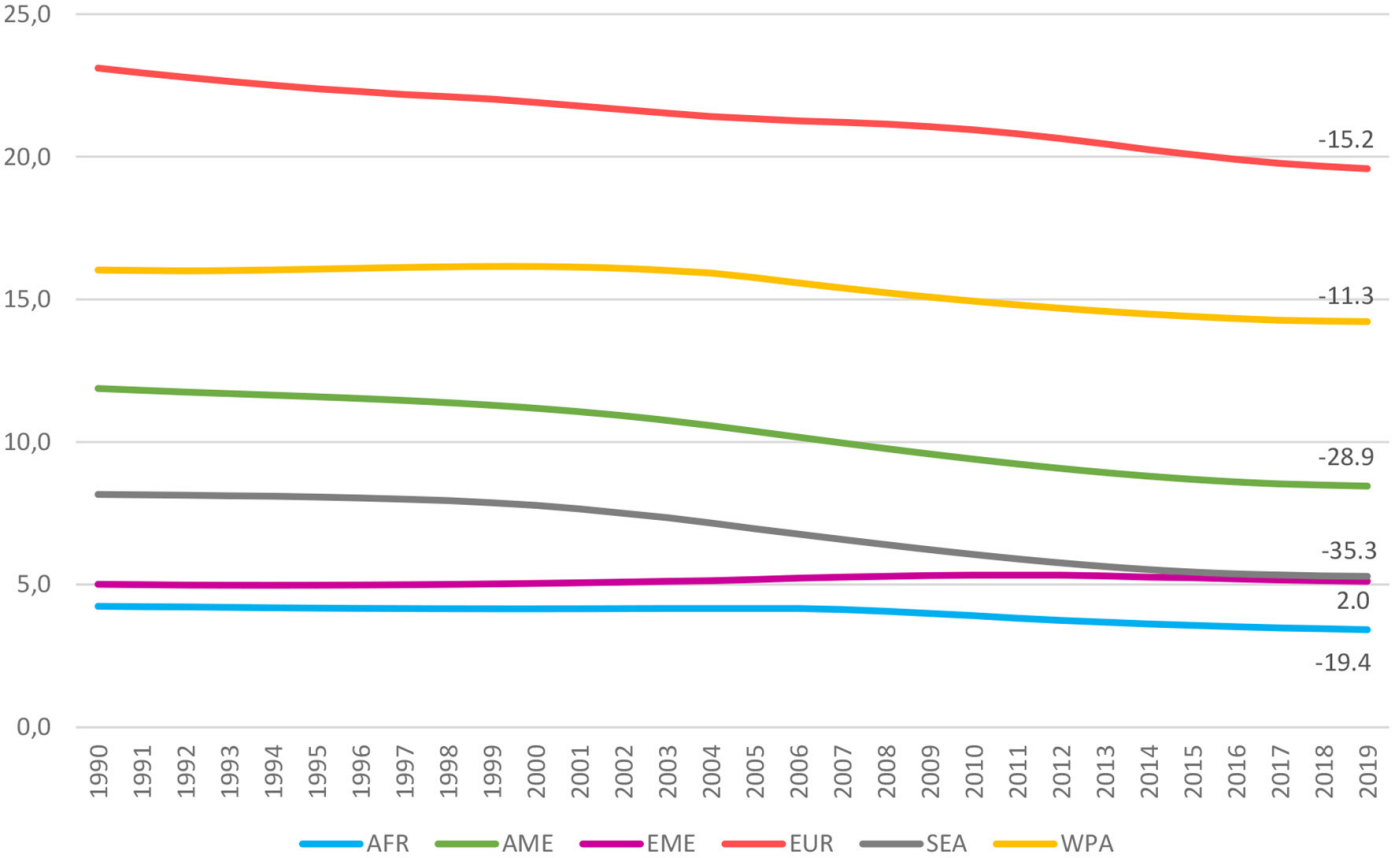
Prevalence of smoking tobacco use among women (by WHO's regions, 1990–2019). Age-stand. rate of smoking tobacco use in the female populations (aged 15 years and older). WHO regions: African (AFR), Americans (AME), Eastern Mediterranean (EME), European (EUR), South-East Asian (SEA), and Western Pacific (WPA).

**Figure 2:**
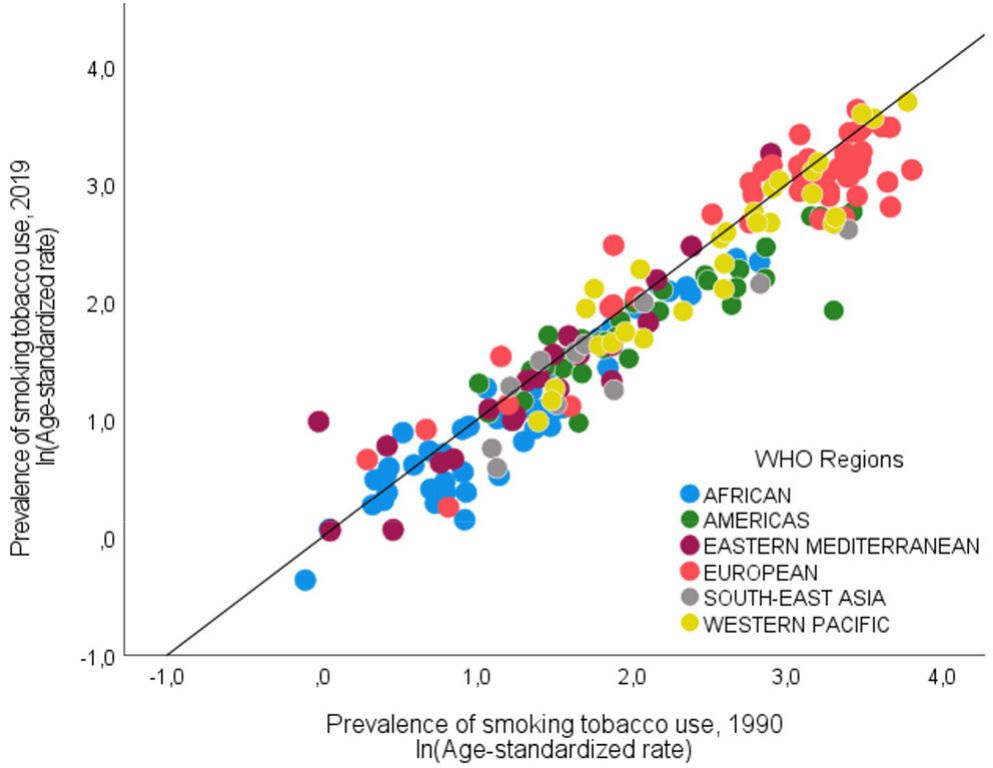
Prevalence of smoking tobacco use among women (by WHO's regions, 1990 vs. 2019). On both axes the natural log of the age stand. rate of smoking tobacco use (female, aged ≥15 years).

The recent worldwide evolution of female smoking prevalence is summarized in Figure 1, where countries are grouped according to the WHO's geographic regions. Since the early 2000 s, the average age-standardized smoking rates have been decreasing in all areas, except for the Eastern Mediterranean region. However, this well-known global overview hides a more mixed country-level picture. As shown in Figure 2—in which the natural logarithm of the prevalence of tobacco smoking in 1990 and 2019 are shown on the horizontal and vertical axes, respectively—countries with an increasing trend in female smoking prevalence (i.e., the dots lying above the 45° line) can be found in every WHO region. Overall, about one-quarter (50/191) of the world's countries have experienced an increase in female smoking prevalence since 1990.

Countries can converge to the same level of smoking prevalence as a result of two different (i.e., increasing or decreasing) long-term paths. Each path has different policy implications for the spread of tobacco-related diseases. Hereafter, we denoted the groups of countries with increasing and decreasing smoking rates as Set *A* and Set *B*, respectively, and we tested the convergence hypothesis in each set. Table 1 reports the results of the Phillips and Sul's (10) *log*-*t* regressions, where the difference between δ_*it*_ and δ_*i*_ is assumed to decline over time, for the set of countries with decreasing and increasing female smoking prevalence, respectively. In both equations, the *t*-statistic is < −1.65. Thus, the null hypothesis of convergence is rejected at a 5% significance level. This evidence provides broad support for the hypothesis that the female smoking prevalence in each group does not converge toward a common level, regardless of the decreasing (or increasing) trend experienced during the last three decades.

**Table 1:** Results of (1) the log-t test, and (2) the ordered logit model.

	***Coeff*.**	***SE***	***T-stat***	***N. of countries***	***N. of years***
**(1) Log-t test**					
Set A: increasing FSP	−0.626	0.014	−44.460	50	30
Set B: decreasing FSP	−0.897	0.005	−172.805	141	30
**(2) Ordered logit model**					
**Set A: increasing FSP**	*Coeff*.	*Robust SE*	*P>|z|*	*Prob > chi2*	*N. of obs*.
Cigarette affordability	0.345	0.056	0.000	0.000	172
	*Odds ratio*	*Robust SE*	*P>|z|*	*Prob > chi2*	
	1.411	0.079	0.000	0.000	
**Set B: Decreasing FSP**	*Coeff*.	*Robust SE*	*P>|z|*	*Prob > chi2*	*N. of obs*.
Cigarette affordability	0.191	0.017	0.000	0.000	541
	*Odds ratio*	*Robust SE*	*P>|z|*	*Prob > chi2*	
	1.210	0.021	0.000	0.000	

*FSP, female smoking prevalence. Affordability: the percentage of per capita GDP required to buy 100 packs of cigarettes*.

**Figure 3:**
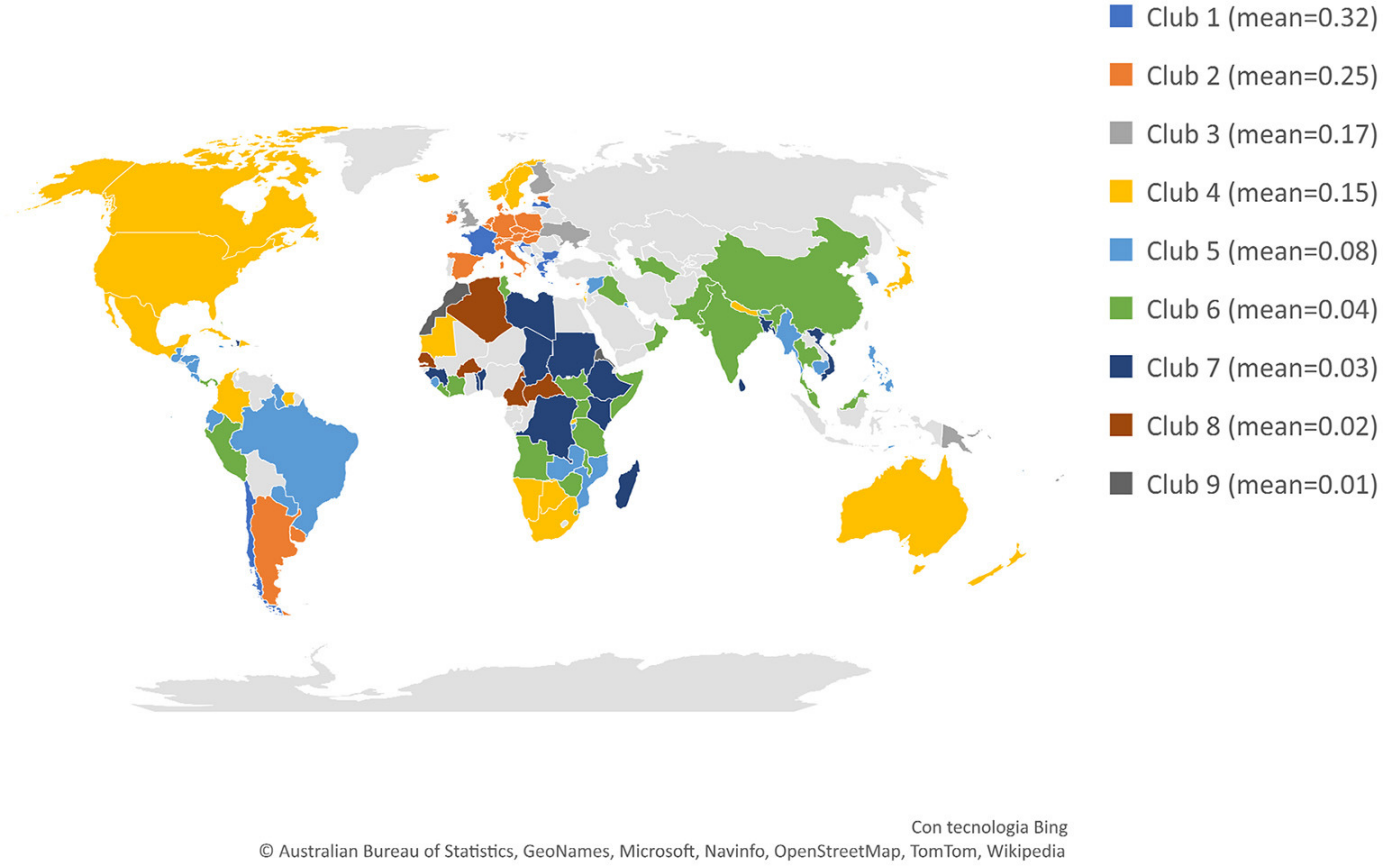
Final clubs (Female. Set B - Countries with decreasing smoking rates, 1990–2019). Countries in light gray are included in the sample with increasing smoking rates or are countries with no data.

However, rejecting the null hypothesis of convergence in each group taken as a whole does not imply that there is no convergence among one or more specific subsets of countries. Phillips and Sul's (10) semi-parametric clustering algorithm enables the endogenous determination of clubs by clustering together those countries that converge to the same steady-state. We found five and nine final clubs among countries with increasing and decreasing smoking prevalence, respectively. Results of the clustering algorithm for the largest set of countries with a declining trend in smoking prevalence (i.e., Set *B*) are displayed in Figure 3, where clubs are ordered from higher to lower values of *FSP*, and the mean values of the smoking prevalence in each club appear beside its color. Results of Set *A*, along with the output of *log t* regressions and the list of countries included in each club for both sets of countries are shown in Supplementary Figure 1 and Supplementary Tables 1, 2 in the Supporting Information File S1.

Turning to the estimations of the ordered logit model, the results (reported at the bottom of Table 1) indicate that cigarette affordability was positively associated with the likelihood of being in a higher club, that is in a club of countries with a low prevalence of female tobacco smoking. These associations, however, can only imply correlation and not causality. Moreover, interpreting the $\hat{\beta }$ coefficients in terms of odds ratios, these results mean that, for a one-unit increase in the relative income price, the probability of falling within a club of countries with a lower female smoking prevalence are 1.41 and 1.21 greater for countries with increasing and decreasing smoking prevalence, respectively.

Finally, we applied the same methodologies to the male smoking prevalence (hereafter, *MSP*) to briefly compare the female and male populations (for conciseness, these results are collected in the Supporting Information File S1). The prevalence of smoking among males increased only in 39 (lower- and lower-middle-income) countries (about 20% of the sample). Again, the null hypothesis of convergence is rejected (at a 5% significance level) in both sets (i.e., increasing and decreasing smoking prevalence), as shown in Supplementary Table 3. Focusing on the most extensive set of countries where smoking rates have decreased, we identified five clubs (Supplementary Figure 2; Supplementary Table 4). Albeit smoking prevalence is, on average, higher among men, club convergence shows similar patterns between the male and female populations, especially in upper-middle and high-income countries. Conversely, among males, cigarette affordability significantly affects the probability of being in a club with a low smoking prevalence only for those countries with a decreasing smoking rate (Supplementary Table 3).

## Discussion

In reviewing the literature, no studies were found on identifying groups of countries in which the female smoking prevalence tends to converge toward similar long-term rates. Hence, the main objective of this brief research paper was to examine convergence in the age-standardized female smoking prevalence among 191 countries worldwide from 1990 to 2019. To this end, we applied Phillips and Sul's (10, 11) time-varying factor model, which allows for individual and transitional heterogeneity, to identify potential convergence clubs.

The evidence indicates that the hypothesis of global convergence is rejected. However, this negative result does not exclude the possibility of convergence within sub-groups of countries. The clustering algorithm successfully identified five and nine final clubs, within countries with increasing and decreasing smoking prevalence, respectively. Unfortunately, these findings are somewhat challenging to interpret. Some groups characterized by a decreasing smoking rate, for example, clubs 2 and 7, are mainly composed of contiguous and relatively similar countries, whereas others (such as club 4, labeled in yellow) contain countries with various cultural backgrounds and socio-economic features. This unexpected heterogeneous composition of some clusters of countries, however, appears to be consistent with earlier research (26, 27), which found that the effectiveness of tobacco control policies has varied significantly worldwide by type of measure and across populations, and showed limited impact among women, especially in low socio-economic environments. A possible explanation for these results may lie in the complex interrelation among the multiple factors that drive smoking habits (28).

Conversely, the results of the ordered logit model add valuable information about the mechanisms at work in the convergence process. Specifically, the positive and statistically significant impact of affordability is consistent with the literature about the association between cigarette affordability and consumption (22) and reaffirms the key role played by price and tax increases in tobacco control globally (29). Moreover, the impact of the *RIP* ratio is greater for the set of countries where smoking rates are rising. This suggests, on the one hand, a large role for fiscal policy in reversing the growing trend in tobacco consumption and, on the other hand, the greater effectiveness of non-fiscal tools (such as anti-smoking campaigns and health-related education programs) in countries where smoking rates are already declining.

These findings are subject to at least two main limitations. The first one is common to similar studies based on GBD estimates (19). GBD data on smoking prevalence come from a large number of nationally representative surveys, modeled using a Spatiotemporal Gaussian Process Regression [a time-series modeling approach that is widely used to estimate time-varying risk factors (7)]. In principle, this procedure might somehow affect convergence results. However, to the best of our knowledge, current literature on convergence analysis does not provide any evidence against the use of such modeled data. The second limitation is about the ordered logit model. Tobacco control measures are key determinants of tobacco consumption (26). Our univariate regression model fails to take into account the impact of regulations on smoking prevalence. This is mainly due to the lack of comprehensive time-series data on the different tobacco control policies for the full period (1990–2019) under investigation. Further research is thus required to analyze both the main determinants of club convergence and the mechanisms that allow countries to transfer from one club to another over time.

## Data Availability Statement

The original contributions presented in the study are included in the article/Supplementary Material, further inquiries can be directed to the corresponding author.

## Author Contributions

FF: conceived and designed the study, analyzed and interpreted the results, and wrote the article. MM: analyzed and interpreted the results and wrote the article. ES: prepared the data, performed the experiments, analyzed and interpreted the results, and wrote the article. All authors read and approved the final manuscript.

## Conflict of Interest

The authors declare that the research was conducted in the absence of any commercial or financial relationships that could be construed as a potential conflict of interest.

## Publisher's Note

All claims expressed in this article are solely those of the authors and do not necessarily represent those of their affiliated organizations, or those of the publisher, the editors and the reviewers. Any product that may be evaluated in this article, or claim that may be made by its manufacturer, is not guaranteed or endorsed by the publisher.
