# Supplementary material for: Testing Club Convergence in Female Smoking Prevalence
Source: Front Glob Womens Health. 2022 Jul 11;3:875813. doi: 10.3389/fgwh.2022.875813 (PMC9309502; doi:10.3389/fgwh.2022.875813)
Supplement: Supplementary file 1 [file Data_Sheet_1.pdf]

Supporting information: File S1.

Supplementary material, including Tables S1, S2, S3, and S4 and Figures S1 and S2.

Table S1. Female. Set B. Decreasing smoking rates. Club composition and log-t test for convergence.

Club 1:<br>9 countries	Bulgaria, Chile, Croatia, France, Greece, Latvia, Malta, Montenegro, and Nauru.	Log(t) Coeff: 0.240<br>T-stat: 2.234
Club 2:<br>21 countries	American Samoa, Andorra, Argentina, Austria, Belgium, Bolivia (Plurinational State of), Cyprus, Czechia, Denmark, Estonia, Germany, Hungary, Ireland, Italy, Luxembourg, Netherlands, Poland, Slovakia, Spain, Switzerland, and Uruguay.	Log(t) Coeff: 0.068<br>T-stat: 0.828
Club 3:<br>9 countries	Fiji, Finland, Palau, Papua New Guinea, Samoa, Tonga, Trinidad and Tobago, Ukraine, and United Kingdom.	Log(t) Coeff: 0.141<br>T-stat: 2.796
Club 4:<br>26 countries	Australia, Bahrain, Botswana, Canada, Colombia, Cuba, Dominica, Dominican Republic, Iceland, Israel, Japan, Maldives, Mauritania, Mauritius, Mexico, Namibia, Nepal, New Zealand, Norway, Rwanda, Seychelles, South Africa, Suriname, Sweden, United States of America, and Venezuela (the Bolivarian Republic of).	Log(t) Coeff: 0.064<br>T-stat: 1.251
Club 5:<br>26 countries	Barbados, Bhutan, Brazil, Brunei Darussalam, Burundi, Cambodia, Costa Rica, Ecuador, Guatemala, Guyana, Honduras, Jamaica, Kuwait, Mozambique, Myanmar, Nicaragua, Paraguay, Philippines, Republic of Korea, Saint Lucia, Sierra Leone, Singapore, Syrian Arab Republic, Timor-Leste, Vanuatu, and Zambia.	Log(t) Coeff: 0.583<br>T-stat: 5.338
Club 6:<br>26 countries	Angola, Armenia, Bahamas, Cabo Verde, China, Comoros, Côte d'Ivoire, Eswatini, India, Iraq, Liberia, Malawi, Malaysia, Oman, Pakistan, Panama, Peru, Saint Kitts and Nevis, Somalia, South Sudan, Thailand, Tunisia, Turkmenistan, Uganda, United Republic of Tanzania, and Zimbabwe.	Log(t) Coeff: 0.146<br>T-stat: 1.899
Club 7:<br>14 countries	Bangladesh, Benin, Chad, the Democratic Republic of the Congo, Ethiopia, Guinea, Haiti, Kenya, Libya, Madagascar, Sri Lanka, Sudan, Togo, and Viet Nam.	Log(t) Coeff: 0.118<br>T-stat: 1.039
Club 8:<br>5 countries	Algeria, Burkina Faso, Cameroon, Central African Republic, and Senegal.	Log(t) Coeff: 1.176<br>T-stat: 4.328
Club 9:<br>2 countries	Eritrea and Morocco.	Log(t) Coeff: 4.683<br>T-stat: 6.673
Not convergent group:<br>3 countries	Gambia, Nigeria, and Tajikistan.	Log(t) Coeff: -2.386<br>T-stat: -8.601

Figure S1. Final clubs (Female. Set A - Countries with increasing smoking rates, 1990-2019).

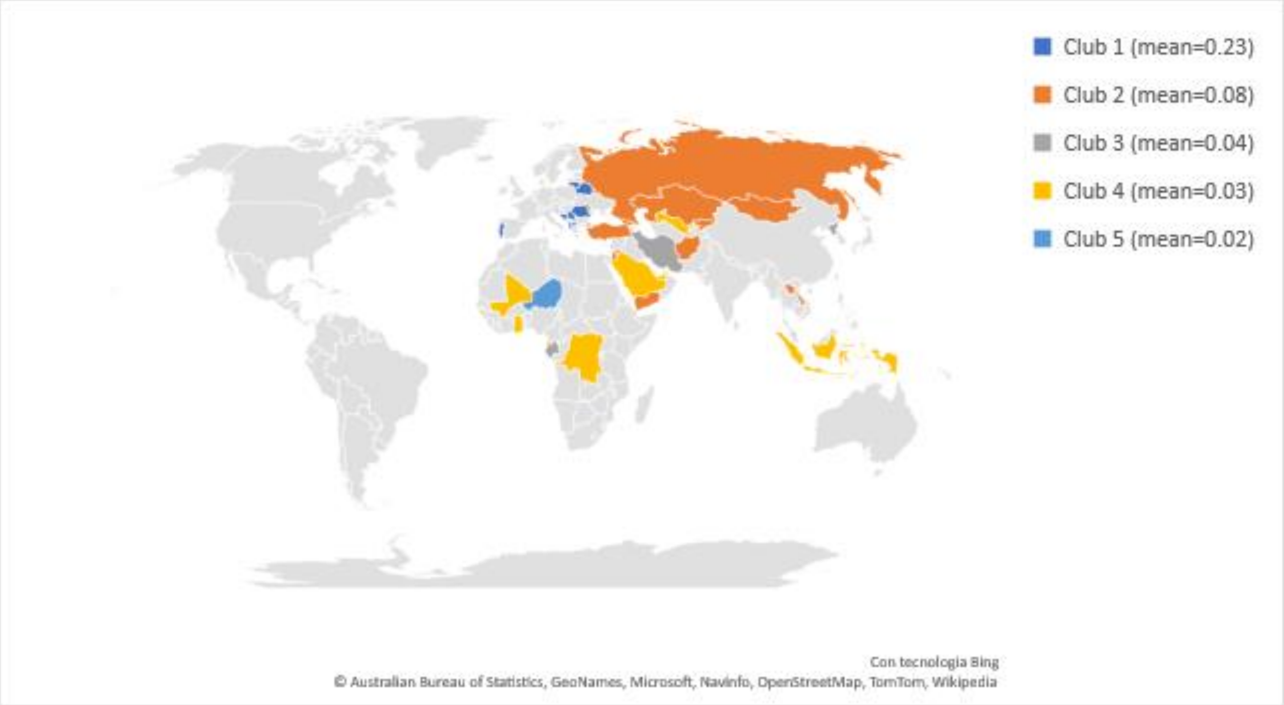

Note: Countries in light grey are included in the sample with decreasing smoking rates or are countries with no data.

Table S2. Female. Set A. Increasing smoking rates. Club composition and log-t test for convergence

Club 1:<br>14 countries	Albania, Belarus, Bosnia and Herzegovina, Kiribati, Lebanon, Lithuania, Micronesia (the Federated States of), North Macedonia, Portugal, Romania, Serbia, Slovenia, Solomon Islands, and Tuvalu.	Log(t) Coeff: 0.191<br>T-stat: 5.406
Club 2:<br>15 countries	Afghanistan, Antigua and Barbuda, Djibouti, El Salvador, Georgia, Jordan, Kazakhstan, Kyrgyzstan, Lao People's Democratic Republic, Marshall Islands, Mongolia, Republic of Moldova, Russian Federation, Turkey, and Yemen.	Log(t) Coeff: -0.005<br>T-stat: -0.263
Club 3:<br>5 countries	Belize, Democratic People's Republic of Korea, Gabon, Grenada, and Iran (the Islamic Republic of).	Log(t) Coeff: 0.277<br>T-stat: 4.556
Club 4:<br>12 countries	Azerbaijan, Congo, Equatorial Guinea, Ghana, Guinea-Bissau, Indonesia, Mali, Qatar, Saint Vincent and the Grenadines, Saudi Arabia, United Arab Emirates, and Uzbekistan.	Log(t) Coeff: -0.019<br>T-stat: -1.181
Club 5:<br>2 countries	Niger, and Sao Tome and Principe.	Log(t) Coeff: 1.172<br>T-stat: 1.282
Not convergent group:<br>2 countries	Egypt, and Lesotho.	Log(t) Coeff: -1.893<br>T-stat: -9.996

Table S3. Male. Results of 1) the log-t test, and 2) the ordered logit model.

			**Log-t test**		
	Coeff.	*SE*	*T-stat*	*N. of countries*	*N. of years*
Set A: Increasing MSP	-0.540	0.003	-170.166	39	30
Set B: Decreasing MSP	-0.940	0.005	-201.434	152	30
			**Ordered logit**		
Set A: Increasing MSP	*Coeff.*	*Robust SE*	*P>|z|*	*Prob > chi2*	*N. of obs.*
Cigarette affordability	0.010	0.031	0.750	0.750	136
	Odds ratio	*Robust SE*	*P>|z|*	*Prob > chi2*	
	1.010	0.031	0.750	0.750	
Set B: Decreasing MSP	*Coeff.*	*Robust SE*	*P>|z|*	*Prob > chi2*	*N. of obs.*
Cigarette affordability	0.024	0.006	0.000	0.000	676
	Odds ratio	*Robust SE*	*P>|z|*	*Prob > chi2*	
	1.024	0.006	0.000	0.000	

Note: *MSP*, male smoking prevalence. Affordability: the percentage of per capita GDP required to buy 100 packs of cigarettes.

Figure S2. Final clubs (Male. Set B - Countries with decreasing smoking rates, 1990-2019).

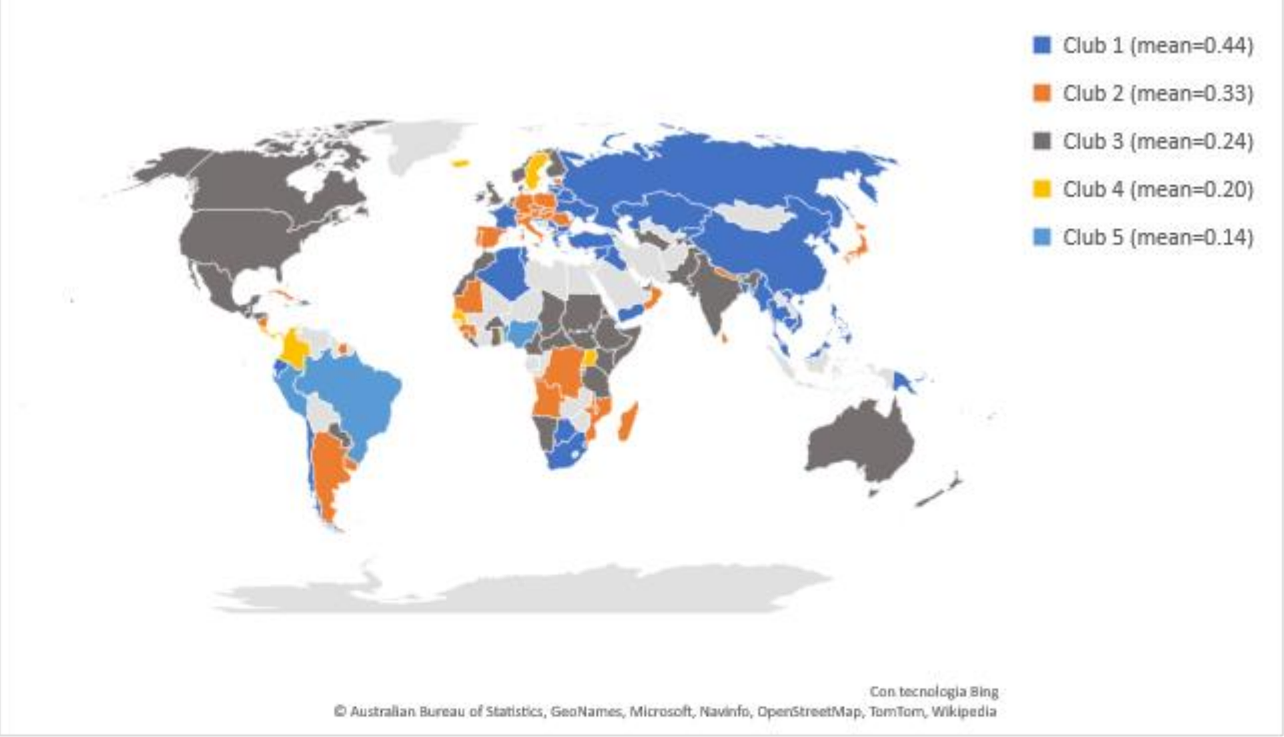

Table S4. Male. Set B. Decreasing smoking rates. Club composition and log-t test for convergence.

Club 1:<br>51 countries	Algeria, American Samoa, Armenia, Azerbaijan, Bahrain, Bangladesh, Belarus, Bolivia (Plurinational State of), Botswana, Bulgaria, Cambodia, Chile, China, Croatia, Cyprus, Democratic People's Republic of Korea, Ecuador, Fiji, France, Greece, Iraq, Kazakhstan, Kyrgyzstan, Latvia, Lithuania, Malaysia, Maldives, Mauritius, Montenegro, Myanmar, Nauru, North Macedonia, Papua New Guinea, Philippines, Republic of Korea, Russian Federation, Samoa, Serbia, Seychelles, Slovenia, South Africa, Syrian Arab Republic, Thailand, Tonga, Trinidad and Tobago, Tunisia, Turkey, Ukraine, Vanuatu, Viet Nam, and Yemen.	Log(t) Coeff: 0.135<br>T-stat: 1.692
Club 2:<br>37 countries	Andorra, Angola, Argentina, Austria, Brunei Darussalam, Cuba, Czechia, the Democratic Republic of the Congo, Estonia, Germany, Guinea, Hungary, Italy, Japan, Kuwait, Madagascar, Malawi, Malta, Mauritania, Mozambique, Nepal, Nicaragua, Oman, Palau, Poland, Portugal, Qatar, Romania, Rwanda, Saint Vincent and the Grenadines, Sierra Leone, Slovakia, Spain, Sri Lanka, Suriname, Switzerland, and Uruguay.	Log(t) Coeff: 0.234<br>T-stat: 2.304
Club 3:<br>50 countries	Australia, Bahamas, Barbados, Belgium, Bhutan, Burkina Faso, Burundi, Cameroon, Canada, Central African Republic, Chad, Comoros, Denmark, Dominica, Dominican Republic, Eritrea, Eswatini, Ethiopia, Finland, Gambia, Ghana, Guatemala, Honduras, India, Ireland, Israel, Jamaica, Kenya, Liberia, Luxembourg, Mexico, Morocco, Namibia, Netherlands, New Zealand, Norway, Pakistan, Paraguay, Saint Lucia, Singapore, Somalia, South Sudan, Sudan, Tajikistan, Turkmenistan, United Arab Emirates, United Kingdom, United Republic of Tanzania, United States of America, and Venezuela (the Bolivarian Republic of).	Log(t) Coeff: -0.010<br>T-stat: -0.192*
Club 4:<br>8 countries	Colombia, Costa Rica, Iceland, Panama, Senegal, Sweden, Togo, and Uganda.	Log(t) Coeff: 0.454<br>T-stat: 2.877
Club 5:<br>6 countries	Benin, Brazil, Cabo Verde, Haiti, Nigeria, and Peru.	Log(t) Coeff: 0.465<br>T-stat: 4.178

Note: *Club 3 is a 'weak club'.
